# Sequencing and analysis of the complete mitochondrial genome of *Blarinella griselda* from China and its phylogenetic analysis

**DOI:** 10.1080/23802359.2020.1715305

**Published:** 2020-02-02

**Authors:** Liu Zhu, Yang Qiao-Jiang, Xu Chun-Yu, Wang Chen, Wang Qing-Qing, Jiang Wen-Jing

**Affiliations:** College of Life Science and Technology, Mudanjiang Normal University, Mudanjiang, P.R. China

**Keywords:** Control region, mitogenome, phylogenetic trees, *Blarinella griselda*

## Abstract

The complete mitogenome sequence of *Blarinella griselda* was determined using long PCR. The genome was 16,947 bp in length and contained 13 protein-coding genes, 2 ribosomal RNA genes, 22 transfer RNA genes, 1 origin of L strand replication and 1 control region. The overall base composition of the heavy strand is A (33.1%), C (22.6%), T (31.6%) and G (12.7%). The base compositions present clearly the A–T skew, which is most obviously in the control region and protein-coding genes. Mitochondrial genome analyses based on MP, ML, NJ and Bayesian analyses yielded identical phylogenetic trees. This study verifies the evolutionary status of *Blarinella griselda* in Soricidae at the molecular level. The mitochondrial genome would be a significant supplement for the *Blarinella griselda* genetic background. The three *Blarinella* species formed a monophyletic group with the high bootstrap value (100%) in all examinations.

In this paper, the complete mitochondrial genome of *Blarinella griselda* was sequenced for the first time on ABI 3730XL using a primer walking strategy and the long and accurate PCR, with five pairs of long PCR primers and with 14 pairs of sub-PCR primers. A muscle sample was obtained from a female *Blarinella griselda* captured from Bijie regions of Wumeng Mountains in Guizhou Province, China (26°24′22″N, 105°44′04″E). The muscle tissue was preserved in 95% ethanol and stored at −75 °C before use. The specimen and its DNA is stored in Animal and Plant Herbarium of Mudanjiang Normal University. The voucher number is GZ201915.

The mitochondrial genome is a circular double-stranded DNA sequence that is 16,947 bp long including 13 protein-coding genes, 2 rRNA genes, 22 tRNA genes, 1 origin of L strand replication and 1 control region. The accurate annotated mitochondrial genome sequence was submitted to GenBank with accession number MN873563. The arrangement of the multiple genes is in line with other Talpidae species (Mouchaty et al. 2000; Nikaido et al. 2003; Cabria et al. 2006; Hou et al. 2016; Xu, Huang et al. 2016; Gutiérrez et al. 2018; Jia et al. 2018) and most mammals (Nikaido et al. 2001; Fontanillas et al. 2005; Meganathan et al. 2012; Yoon et al. 2013; Xu et al. 2012, 2013; Kim et al. 2013, 2017; Huang et al. 2014, 2016; Xu et al. 2016; Liu et al. 2016, 2018; Liu, Dang, et al. 2019; Liu, Qin, et al. 2019; Liu, Tian, Jin, Dong, et al. 2017; Liu, Tian, Jin, Jin, et al. 2017; Liu, Wang, et al. 2017; Jin et al. 2017).

The control region of *Blarinella griselda* mitochondrial genome was located between the tRNA-Pro and tRNA-Phe genes, and contains only promoters and regulatory sequences for replication and transcription, but no structural genes. Three domains were defined in the large mole mitochondrial genome control region (Zhang et al. 2009): the extended termination-associated sequence (ETAS) domain, the central conserved domain (CD) and the conserved sequence block (CSB) domain.

The total length of the protein-coding gene sequences was 11,416 bp. Most protein-coding genes initiate with ATG except for ND2, ND3 and ND5, which began with ATA or ATT. Six protein-coding genes terminated with TAA whereas the Cyt b gene terminated with AGA. The incomplete stop codons (T– – or TA–) were used in COX3, ATP6, ND3, and ND4. A strong bias against A at the third codon position was observed in the protein-coding genes. The frequencies of CTA (Leu), ATT (Ile), TTA (Leu) and ATA (Met) were higher than those of other codons. The length of tRNA genes varied from 59 to 75 bp.

Most *Blarinella griselda* mitochondrial genes were encoded on the H strand, except for the ND6 gene and eight tRNA genes, which were encoded on the L strand. Some reading frame intervals and overlaps were found. One of the most typical was between ATP8 and ATP6. The L-strand replication origin (OL) was located within the WANCY region containing five tRNA genes (tRNATrp, tRNA-Ala, tRNA-Asn, tRNA-Cys, tRNA-Tyr). This region was 36 bp long and had the potential to fold into a stable stem-loop secondary structure. The total base composition of *Blarinella griselda* mitochondrial genome was A (33.1%), C (22.6%), T (31.6%) and G (12.7%). The base compositions clearly present the A-T skew, which was most obviously in the control region and proteincoding genes.

In order to explore the evolution of Insectivora shrews which include Soricidae and Talpidae, especially the evolution of genus *Blarinella* from China, here, we investigate the molecular phylogenetics of Chinese *Blarinella griselda* using complete mitochondrial genome sequence of 37 species. All sequences generated in this study have been deposited in the GenBank (Figure 1).

**Figure 1.**
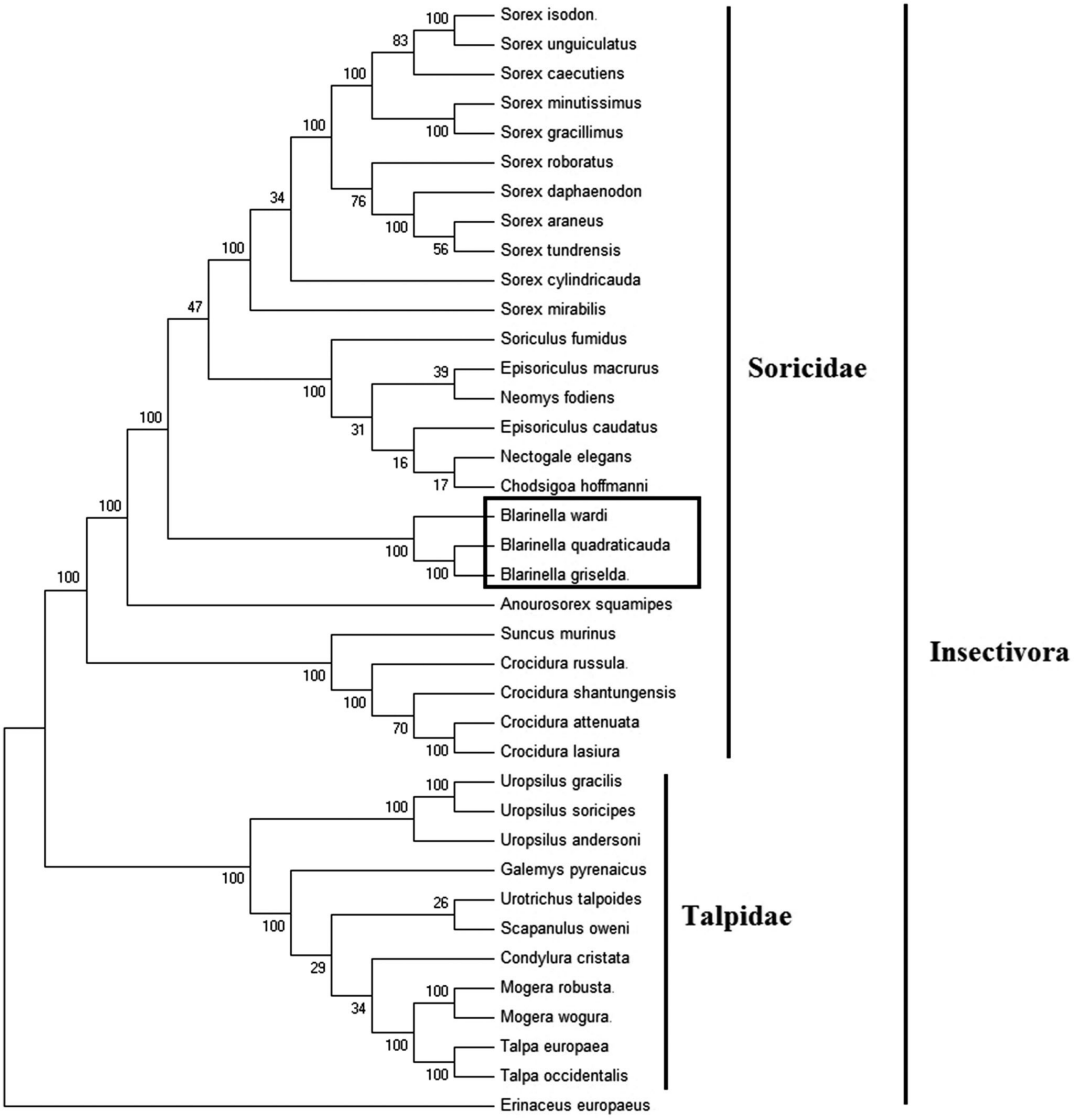
Phylogenetic tree generated using the Maximum Parsimony method based on complete mitochondrial genomes. *Chodsigoa hoffmanni* (MK940327), *Crocidura lasiura* (KR007669), *Crocidura shantungensis* (JX968507), *Crocidura attenuata* (KP120863), *Crocidura russula* (AY769264), *Episoriculus macrurus* (KU246040), *Episoriculus caudatus* (KM503097), *Neomys fodiens* (KM092492), *Nectogale elegans* (KC503902), *Anourosorex squamipes* (KJ545899), *Blarinella quadraticauda* (KJ131179), *Blarinella wardi* (MF125692), *Suncus murinus* (KJ920198), *Soriculus fumidus* (AF348081), *Sorex araneus* (KT210896), *Sorex cylindricauda* (KF696672), *Sorex unguiculatus* (AB061527), *Sorex tundrensis* (KM067275), *Sorex caecutiens* (MF374796), *Sorex roboratus* (KY930906), *Sorex isodon* (MG983792), *Sorex gracillimus* (MF426913), *Sorex mirabilis* (MF438265), *Sorex daphaenodon* (MK110676), *Sorex minutissimus* (MH823669), *Talpa europaea* (Y19192), *Urotrichus talpoides* (AB099483), *Uropsilus soricipes* (JQ658979), *Uropsilus gracilis* (KM379136), *Mogera wogura* (AB099482), *Mogera robusta* (MK431828), *Condylura cristata* (KU144678), *Galemys pyrenaicus* (AY833419), *Scapanulus oweni* (KM506754), *Talpa occidentalis* (MF958963), *Uropsilus andersoni* (MF280389), *Erinaceus europaeus* (NC002080).

Mitochondrial genome analyses based on MP, ML, NJ and Bayesian analyses yielded identical phylogenetic trees, indicating a close phylogenetic affinity of shrews. The phylogram obtained from Maximum Parsimony method is shown in Figure 1. It shows that two major phyletic lineages were present in Insectivora: Soricidae and Talpidae. Soricidae comprised *Blarinella griselda, Blarinella wardi, Blarinella quadraticauda*, *Chodsigoa hoffmanni, Crocidura lasiura*, *Crocidura shantungensis*, *Crocidura attenuata*, *Crocidura russula*, *Episoriculus macrurus*, *Episoriculus caudatus*, *Neomys fodiens*, *Nectogale elegans*, *Anourosorex squamipes*, *Soriculus fumidus*, *Suncus murinus*, *Sorex araneus*, *Sorex tundrensis*, *Sorex caecutiens*, *Sorex roboratus*, *Sorex isodon*, *Sorex gracillimus*, *Sorex mirabilis*, *Sorex cylindricauda, Sorex unguiculatus, Sorex daphaenodon* and *Sorex minutissimus* was supported by bootstrap values of 100%. Talpidae comprised *Talpa europaea*, *Urotrichus talpoides*, *Mogera wogura*, *Condylura cristata*, *Uropsilus soricipes*, *Mogera robusta*, *Galemys pyrenaicus*, *Uropsilus gracilis, Talpa occidentalis, Uropsilus andersoni* and *Scapanulus oweni* was supported by bootstrap values of 100%. This study verifies the evolutionary status of *Blarinella griselda* in Soricidae at the molecular level. The mitochondrial genome would be a significant supplement for the *Blarinella griselda* genetic background. The three *Blarinella* species formed a monophyletic group with the high bootstrap value (100%) in all examinations.
